# Patients’ and caregivers’ perceptions of bariatric surgery: A France and United States comparative infodemiology study using social media data mining

**DOI:** 10.3389/fdgth.2023.1136326

**Published:** 2023-04-18

**Authors:** Sébastien Czernichow, Nathalie Rassy, Joelle Malaab, Paul Loussikian, Adel Mebarki, Mickail Khadhar, Tigran Poghosyan, Guy Fagherrazi, Claire Carette, Stéphane Schück, Claire Rives-Lange

**Affiliations:** ^1^ Assistance Publique-Hôpitaux de Paris (AP-HP), Service de Nutrition, Centre Spécialisé Obésité, Hôpital Européen Georges Pompidou, Paris, France; ^2^ Université Paris Cité, Paris, France; ^3^ INSERM, UMR1153, Epidemiology and Biostatistics Sorbonne Paris Cité Center (CRESS), METHODS Team, Paris, France; ^4^ Kap Code, Paris, France; ^5^ Assistance Publique-Hôpitaux de Paris (AP-HP), Service de chirurgie digestive, Hôpital Bichat, Paris, France; ^6^ Deep Digital Phenotyping Research Unit, Department of Precision Health, Luxembourg Institute of Health, Strassen, Luxembourg; ^7^ Assistance Publique-hôpitaux de Paris (AP-HP), Centre d’investigation clinique, Inserm 1418, Hôpital Européen Georges Pompidou, Paris, France

**Keywords:** bariatric (weight loss) surgery, social media, patients, caregiver, perception, infodemiology

## Abstract

**Background:**

People are conversing about bariatric surgery on social media, but little is known about the main themes being discussed.

**Objective:**

To analyze discussions regarding bariatric surgery on social media platforms and to establish a cross-cultural comparison of posts geolocated in France and the United States.

**Methods:**

Posts were retrieved between January 2015 and April 2021 from general, publicly accessed sites and health-related forums geolocated in both countries. After processing and cleaning the data, posts of patients and caregivers about bariatric surgery were identified using a supervised machine learning algorithm.

**Results:**

The analysis dataset contained a total of 10,800 posts from 4,947 web users in France and 51,804 posts from 40,278 web users in the United States. In France, post-operative follow-up (*n* = 3,251, 30.1% of posts), healthcare pathways (*n* = 2,171, 20.1% of the posts), and complementary and alternative weight loss therapies (*n* = 1,652, 15.3% of the posts) were among the most discussed topics. In the United States, the experience with bariatric surgery (*n* = 11,138, 21.5% of the posts) and the role of physical activity and diet in weight-loss programs before surgery (*n* = 9,325, 18% of the posts) were among the most discussed topics.

**Conclusion:**

Social media analysis provides a valuable toolset for clinicians to help them increase patient-centered care by integrating the patients’ and caregivers’ needs and concerns into the management of bariatric surgery.

## Introduction

It is estimated that over 650 million individuals are living with obesity (1). This figure will expand to over one billion in 2030 (2, 3). Bariatric surgery is an established treatment for severe obesity and its comorbidities. Due to the increased number of patients with obesity, it is projected that the number of bariatric surgeries will continue to increase (4–6). These weight-loss surgical procedures will improve obesity-related comorbidities (7).

Bariatric surgery patients face multi-faceted medical, nutritional, and psychological issues (8), and acknowledging their needs and concerns is necessary for effective patient assessment in both clinical practice and research. Qualitative methods are traditional methods that are widely used to establish an in-depth thematic analysis of patients’ perceptions but are often conducted on a small sample scale. Infodemiology study of social media offers a valuable addition to qualitative studies which allows identifying a broad spectrum of patients’ perceptions more spontaneously than traditional methods (9–11).

The use of social networking sites and peer-to-peer virtual communities to discuss health conditions and treatment experiences has gained worldwide popularity over the last decade. Individuals with obesity and bariatric surgery patients are more often turning to social media to look for information and support, as well as to share their experiences (12–16). Despite evidence suggesting that people are conversing about bariatric surgery on social media, little is known about the main themes being discussed. To narrow this gap, the present study aimed to analyze the content of web-based discussions on social media and to establish a cross-cultural comparison of discussions from accounts created in France and the United States (US).

## Materials and methods

### Study design and population

This is a retrospective study using data from social media geolocated in France and the US, posted by patients who had undergone bariatric surgery and their caregivers.

### Data extraction

Posts were retrieved between January 2015 and April 2021 from general, publicly accessed sites (e.g., Twitter), and health-related forums (e.g., Doctissimo, in France). Due to restricted data access and closed groups, Facebook, Instagram, and Whatsapp were excluded. To identify pertinent posts, an extraction query featuring relevant keywords was developed. Keywords associated with bariatric surgery were identified in French (i.e., chirurgie bariatrique, anneau gastrique, sleeve, bypass, chirurgie de l’obésité) and English (i.e., bypass, bariatric, sleeve), and included within the extraction query. Using the Brandwatch® extractor (Cision Ltd.), we identified and gathered all publicly available messages that contained one of the required keywords, along with their associated metadata (e.g., author and publication date). When applicable, various spellings of a keyword were considered in the extraction query to include all versions an internet user might spell a keyword.

### Preprocessing and cleaning of data

Posts were harmonized under the same format to achieve a synchronized version of the dataset and a smoother cleaning and analysis process. This step entailed switching all characters in the posts to lower case format and removing all accents and apostrophes from words. The cleaning process consisted of establishing a list of exclusion criteria: duplicates, posts from sources deemed unsafe or irrelevant (e.g., advertising websites, forums related to cars, pets, or animals), posts containing 5 words or less, and posts exceeding 10,000 characters were removed. Posts with less than 5 words do not contain enough information to be effectively interpreted and posts exceeding 10,000 characters are rare and were excluded from the analysis dataset due to their excessive processing time. To analyze the volume of posts per keyword, a presence step was applied: keywords were automatically identified within the dataset via an automatic search, and subsequently matched to their respective posts. Posts of patients and caregivers were identified *via* a supervised machine learning algorithm using lexical fields suggestive of a patient’s or caregiver’s personal experiences, medical conditions, and the pronouns featured in a message [e.g., “I have (EXTRACTION TERM)”]. The algorithm was, therefore, able to identify relevant posts and compute the probability of whether the user is a patient or a caregiver. In order to further advance the filtering process, a supervised machine learning algorithm was applied to identify posts associated with patients’ or caregivers’ experiences. This algorithm was previously developed using a training set of 12,330 messages related to different health domains (dermatology, tobacco use, oncology, among others). The method consists, in a pipeline, of two XGBoost classifiers (one for caregivers’ experiences, one for patients’), applied successively. This method allowed us to identify if a post belonged to a patient, a caregiver, or neither. Both classifiers are based on features combining pronouns and lexical fields describing relatives and pathologies. The caregiver classifier was trained on the whole training set and the patient classifier on posts from non-caregivers. Evaluation of performances yielded respective F1 score (a measure of accuracy combining precision and recall) of 88% and 87% for the caregiver and patient classifier. The algorithm was trained and tested on a sample of 12,330 posts. Its ability to detect patients generated the following performance results: an accuracy of 81%, a F1-score of 87%, a sensibility of 88%, a specificity of 66%, and a precision of 85%. As for the detection of caregivers, the algorithm yielded: an accuracy of 85%, a F1-score of 88%, a sensibility of 94%, a specificity of 72%, and a precision of 83%.

### Data analysis

The main discussion themes among patients and caregivers were identified using Biterm Topic Modeling. The latter is a natural language processing, text mining approach that clusters similar texts based on common topics (17). To eliminate subjective biases, Biterm Topic Modeling was applied without prior knowledge of existing topics. This approach automatically aggregates posts under different categories, in descending order of frequency. Each category consists of a list of its most recurrent words, allowing us to interpret and identify its related discussion topic.

## Results

**Figure 1:**
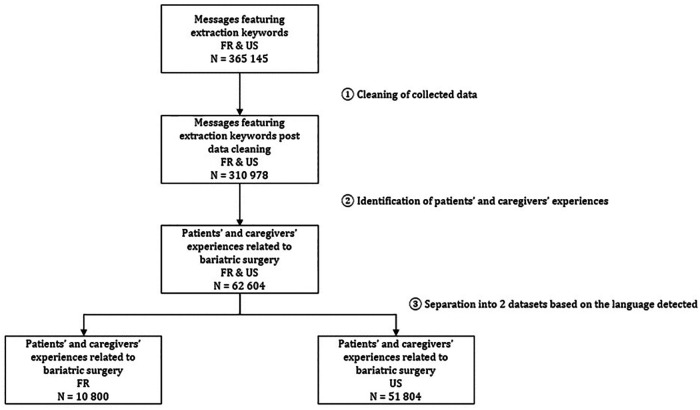
Flowchart presenting posts extraction from social media geolocated in France and the US.

A total of 3,65,145 posts were initially retrieved written by a range of internet users discussing bariatric surgery. Data cleaning and processing allowed us to identify patients’ and caregivers’ discussions. As a result, the analysis dataset contained a total of 10,800 posts in France and 51,804 posts in the US (Figure 1). The number of significant keywords in retrieved messages is displayed in the multimedia Appendix S1.

**Figure 2:**
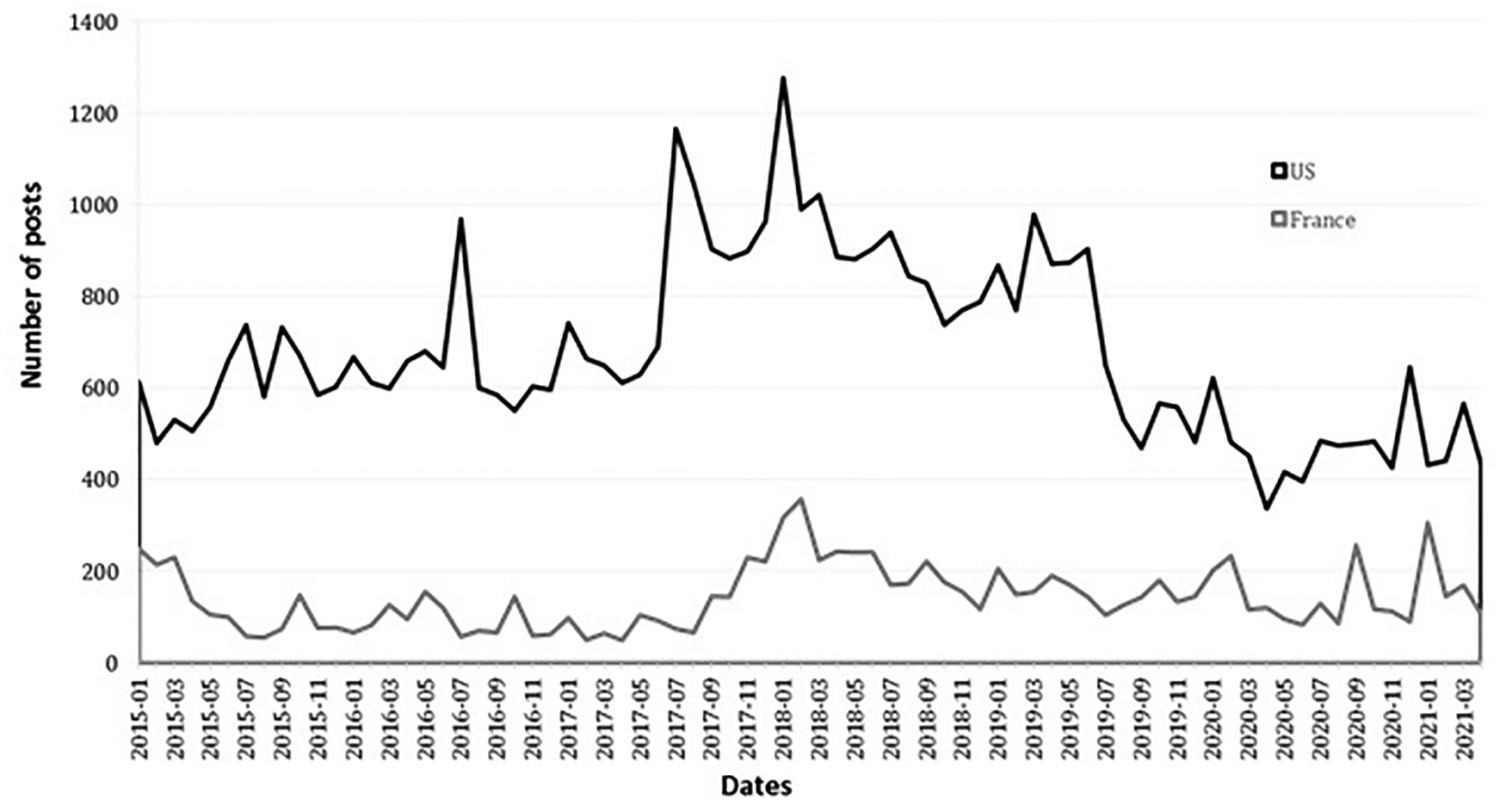
Temporal trend of the number of posts on bariatric surgery extracted between January 2015 and March 2021 from social media geolocated in France and the US.

The number of web users was higher in the US (*n* = 40,278) than in France (*n* = 4,947) during the period of analysis. Figure 2 shows the temporal evolution of the number of posts extracted in both countries.

Posts originated from 24 different social media platforms in France and the US (Multimedia Appendix S2). In France, Twitter was the main source of data (*n* = 5,674, 53.4% of posts and *n* = 3,032, 62.7% of web users) followed by health-related forums such as Doctissimo (*n* = 3,403, 32% of posts, *n* = 1,108, 22.9% of web users) and Aufeminin (*n* = 714, 6.7% of posts, *n* = 370, 7.7% of web users). Similarly in the US, posts mainly stemmed from Twitter (*n* = 11,902, 27.7% of posts and *n* = 8,700, 26.5% of web users). Reddit also provided an important volume of data (*n* = 7,561, 17.6% of posts and *n* = 5,725, 17.4% of users) followed by websites that are dedicated to bariatric surgery and obesity such as Bariactricpal (*n* = 6,017, 14% of posts and *n* = 5,837, 17.8% of web users), Obesityhelp (*n* = 3,957, 9.2% of posts and *n* = 2,516, 7.7% of web users), and Myfitnesspal (*n* = 3,254, 7.6% of posts and *n* = 2,232, 6.8% of web users).

The major themes and topics of bariatric surgery patients and caregivers differed between the two countries (Table 1). In France, discussions centered on patients, mainly about their post-operative follow-up (*n* = 3,251, 30.1% of posts). Discussions also involved bariatric patients’ healthcare pathways (*n* = 2,171, 20.1% of the posts). Many patients were interested in complementary and alternative weight loss therapies (*n* = 1,652, 15.3% of the posts), discussing their social life (*n* = 788, 7.3% of the posts), and searching for hospitals and clinics performing bariatric surgery (*n* = 691, 6.4% of the posts). In the US, posts mainly included caregivers’ discussions. Caregivers discussed the experience of their families or friends with bariatric surgery (*n* = 11,138, 21.5% of the posts). Discussions also focused on physical activity and diet in weight-loss programs before bariatric surgery (*n* = 9,325, 18% of the posts) and on the effectiveness of sleeve gastrectomy (*n* = 8,548, 16.5% of the posts). As seen in France, posts in the US also included the topic of post-operative experience and follow-up (*n* = 5,180, 10% of the posts).

**Table 1:** Topic modeling.

	Posts, *n* (%)
France	
Bariatric surgery and post-operative follow-up	3,251 (30.1)
Healthcare pathway for prospective bariatric surgery patients	2,171 (20.1)
Complementary and alternative weight loss therapies	1,652 (15.3)
Social/relational impact of bariatric surgery on patients’ and caregivers’ lives	788 (7.3)
Information about bariatric public hospitals and private clinics	691 (6.4)
Management of comorbidities	594 (5.5)
Testimonials about the surgeons	497 (4.6)
Food and diets following surgery	486 (4.5)
Women’s health	421 (3.9)
Others	248 (2.3)
United States	
Testimonials from caregivers about the journey of family or friends who have had surgery	11,138 (21.5)
Exercise, food, and diet following surgery	9,325 (18.0)
Discussions about sleeve gastrectomy	8,548 (16.5)
Post-operative experience and follow-up	5,180 (10.0)
Patients’ emotional journey	4,403 (8.5)
Insurance issues with bariatric surgery	3,419 (6.6)
Comorbidities that motivated bariatric surgery	3,264 (6.3)
Impact on daily activities	3,264 (6.3)
Medical treatment of postoperative complications and comorbidities	1,658 (3.2)
Support groups	1,140 (2.2)
Others	466 (0.9)

## Discussion

### Principal findings

To our knowledge, this study is the first to show that bariatric surgery is actively discussed on social media, with 62,604 posts from 45,225 different web users. France and the United States are in the top three countries in terms of volume of bariatric surgery procedures per year. Interestingly, the number of web users was greater in the US than in France. On one hand, this is likely because the US is one of the highest social network user rates in the world with approximately 80% of the US population having a social networking profile (2). On the other hand, this may be a consequence of the relatively higher proportion of individuals with obesity and the larger number of bariatric surgeries in the US, in comparison with France (18, 19). The rate of weight stigma is significantly higher in the US compared to France (20) and the anonymity of internet use may explain the large number of American web users discussing bariatric surgery. Because of social stigmatization and discrimination (20), patients with obesity may have particular use of health care (21) and may turn to social media to interact with others and share experiences (22).

The evidence of the present infodemiology study found that bariatric surgery is an actively discussed topic in French and American social media. A rigorous text mining protocol identified some dissimilarities in major topics discussed in both countries. Patients in France mainly discuss post-operative follow-up, healthcare pathways, and behavioral therapies for weight loss. However, caregivers in the US share the journey of their family and friends, discuss weight-loss programs before surgery, and request information about bariatric surgery. Despite some dissimilarities, there are similarities in several topics between the two countries. Web users in the US and France express their concerns about nutritional and psychological problems after bariatric surgery and emphasize the importance of postoperative follow-up from health professionals. Furthermore, web users of both countries discuss several techniques of bariatric surgery. In France, there is a focus on complementary and alternative weight loss therapies among patients considering non-invasive surgeries. In the US, sleeve gastrectomy is a much-discussed technique. Many patients use this technique and observe significant variable effectiveness, which encourages them to share their experiences with others.

Identifying discussion topics on social media serve as an essential complementary source of health data because they are “real-life” data, their collection does not require a complex information-gathering device, and they are immediately accessible and analyzable. Discussing medical conditions and treatment as well as sharing experiences on social media have been largely investigated in patients taking medications such as neuroleptics and paracetamol or suffering from atopic dermatitis, irritable bowel syndrome, and cancer (23–28). Our analysis showed the popularity of bariatric surgery discussions on social media. We focused on general and publicly accessed sites as well as health-related forums. The results from other studies using other media sources such as Facebook and Instagram demonstrated that giving and seeking recommendations, sharing nutrition-related advice, discussing post-bariatric changes, and sharing experiences after surgery are the most common topics (13, 29, 30). In a previous study, a research group used free word association network analysis to examine the mental and emotional representation of weight loss in bariatric surgery patients on Facebook (31). The results indicated that bariatric surgery patients commonly think about weight loss in two distinct ways: they may emphasize the potential positive outcomes of losing weight, or they may focus more on the process of how to achieve it.

Our infodemiologic research has identified several main fields of online discussions related to bariatric surgery. By addressing these areas, bariatric centers can improve the quality of care they provide and better meet the needs of their patients. Here are some recommendations for bariatric centers on how they can improve in these areas to better accommodate the needs of their patients. First, bariatric centers should provide patients with clear and accurate information about the different types of bariatric surgery, the potential risks and benefits, and the lifestyle changes required for long-term success. Second, they should offer ongoing education and support to patients, both before and after surgery by including nutrition education, behavioral counseling, and support groups, Third, they should monitor patients’ outcomes, provide ongoing support and follow-up care, and recognize the importance of psychological and emotional support for patients before and after surgery. Finally, bariatric centers should focus on engaging with their patients through various online platforms in order to better understand their needs. This can include creating user-friendly websites, maintaining an active social media presence, and providing a secure and easily accessible patient portal.

### Limitations of the study

The present study is the first to advance the limited cross-country literature on patients’ and caregivers’ perceptions of bariatric surgery, but some limitations should be considered. Firstly, patients’ or caregivers’ characteristics such as age, socioeconomic status, comorbidities, and severity of obesity were not included in the present study. Secondly, discussions on private or restricted patient groups and applications such as WhatsApp were not analyzed. Thirdly this study might be subject to a selection bias as it excludes individuals who do not have access to the internet and older generations who do not express their concerns on social media (32). This study has relied on the retrospective reports of family members or friends, increasing the likelihood of recall bias. Also, a measurement bias may be associated with semantic analysis and automated language processing (33, 34). At last, the analysis of data obtained from social networks is sometimes incomplete and may not be clarified which may lead to some misinterpretations (35–37).

## Conclusion

Our infodemiology approach has shown that identifying discussion topics on social media is feasible and can provide a possible toolset for researchers and clinicians to capture broad-spectrum patient and caregiver perceptions toward bariatric surgery.

## Data Availability

The code analyzed in this study is subject to the following licenses/restrictions: Brandwatch, which is a data crawling specialist [Copyright © 2023 Brandwatch. All Rights Reserved]; xgboost algorithm [Apache License (==2.0)] to classify messages (patients, caregivers or respondents); this algorithm is coded in R [KAP CODE INTELLECTUAL PROPERTY]; Topic modelling to analyze the topics of discussion; the topic modelling was based on a CRAN Package called BTM [Apache License (==2.0)] that we adapted [KAP CODE INTELLECTUAL PROPERTY]; All these codes were written in R and used in Rstudio IDE [GNU AFFERO GENERAL PUBLIC LICENSE – VERSION 3]. Requests to access these datasets should be directed to Kapcode; *paul.loussikian@kapcode.fr* and *adel.mebarki@kapcode.fr*.
