# Supplementary material for: Patients’ and caregivers’ perceptions of bariatric surgery: A France and United States comparative infodemiology study using social media data mining
Source: Front Digit Health. 2023 Apr 18;5:1136326. doi: 10.3389/fdgth.2023.1136326 (PMC10151923; doi:10.3389/fdgth.2023.1136326)
Supplement: Supplementary file 1 [file Table1.docx]

| **Multimedia appendix 1. Number of keywords in messages in both countries** | |  |
| --- | --- | --- |
| **Forums** | **Number** | |
| **France*** |  | |
| BARIATRIC SURGERY | 2788 | |
| GASTRIC BANDNG | 2596 | |
| SLEEVE | 2374 | |
| BYPASS | 2001 | |
| SURGERY FOR OBESITY | 1041 | |
| **United States*** |  | |
| BYPASS | 22082 | |
| BARIATRIC | 17926 | |
| SLEEVE | 11796 | |

| **Multimedia appendix 2. List of the social network or websites** | | |
| --- | --- | --- |
| **Forums** | **Posts**  **n (%)** | **Web users**  **n (%)** |
| **France*** |  |  |
| TWITTER | 5674 (53.4%) | 3032 (62.7%) |
| DOCTISSIMO | 3403 (32.0%) | 1108 (22.9%) |
| AU FEMININ | 714 (6.7%) | 370 (7.7%) |
| BABYCENTER.FR | 224 (2.1%) | 129 (2.7%) |
| OBESITE-87.COM | 201 (1.9%) | 1 (0%) |
| **United States*** |  |  |
| TWITTER | 11902 (27.7%) | 8700 (26.5%) |
| REDDIT | 7561 (17.6%) | 5725 (17.4%) |
| BARIACTRICPAL.COM | 6017 (14.0%) | 5837 (17.8%) |
| OBESITYHELP.COM | 3957 (9.2%) | 2516 (7.7%) |
| MYFITNESSPAL.COM | 3254 (7.6%) | 2232 (6.8%) |
| THINNERTIMESFORUM.COM | 2030 (4.7%) | 1942 (5.9%) |
| BABYCENTER.COM | 1872 (4.4%) | 1383 (4.2%) |
| GASTRICSLEEVE.COM | 1196 (2.8%) | 811 (2.5%) |
| BARIATRICFACTS.ORG | 950 (2.2%) | 446 (1.4%) |
| CITY-DATA.COM | 485 (1.1%) | 314 (1.0%) |
| LIPSTICKALLEY.COM | 443 (1.0%) | 345 (1.1%) |
| * list of social network or websites that have a percentage of posts less than 1%  In France: carenity.com, e-sante, journaldesfemmes.fr, journaldesfemmes.com, commentcamarche.net, psychologies, multiesthetique.fr, dailymotion, forum-ecigarette.com, forumlyme.com, ligue contre le cancer, baclofene.com, futura-sciences, japancar.fr, madmoizelle.com, dna.fr, entrepatients.net, magic maman, yabiladi  In United States: disboards.com, weightwatchers.com, dcurbanmom.com, myproana.com  inspire.com, wlsurgery.com, medhelp.org, delphiforums.com, cafemom.com, breast cancer, straightdope.com, diabetesdaily.com, lowcarber.org | | |
